# Tumor‐derived endomucin promotes colorectal cancer proliferation and metastasis

**DOI:** 10.1002/cam4.5055

**Published:** 2022-08-15

**Authors:** Qi Huang, Xue‐mei Li, Jing‐ping Sun, Yan Zhou

**Affiliations:** ^1^ NHC Key Laboratory of Nuclear Technology Medical Transformation, Mianyang Central Hospital, School of Medicine University of Electronic Science and Technology of China Mianyang Sichuan PR China; ^2^ The First Affiliated Hospital of Chengdu Medical College, Clinical Medical College Chengdu Medical College Chengdu Sichuan China

**Keywords:** colorectal cancer, endomucin, metastasis, proliferation

## Abstract

**Background:**

Endomucin (EMCN) is a type I transmembrane glycoprotein and a mucin‐like component of the endothelial cell glycocalyx. The mechanism of EMCN action in colorectal cancer (CRC) remains unclear.

**Aims:**

Our aim was to explore the role of EMCN in the progression of CRC.

**Materials & Methods:**

We examined EMCN expression in CRC tissues and normal para‐carcinoma tissues. The function and mechanisms of EMCN were checked in CRC cell lines and in mouse xenograft. Additionally, we used co‐immunoprecipitation and mass spectrometry to identify the potential EMCN‐binding proteins. Functional annotation analysis showed where these genes were enriched.

**Results:**

We found that EMCN was overexpressed in tumor tissues compared with that in normal para‐carcinoma tissues. We also found that overexpression of EMCN induced CRC proliferation and metastasis both in vitro and in vivo. EMCN knockdown prevents epithelial‐mesenchymal transition in vitro. We identified 178 potential EMCN‐binding partners. Furthermore, functional annotation analysis indicated that these genes were considerably enriched in carcinogenic‐related functions and pathways. Collectively, the identification of EMCN‐binding partners enhanced our understanding of the mechanism of EMCN‐mediated malignant phenotypes, and this research may provide valuable insights into the molecular mechanisms underlying CRC.

**Conclusion:**

Tumor‐derived endomucin promotes colorectal cancer proliferation and metastasis. We identified 178 EMCN‐binding proteins and initially screened three potential EMCN‐interacting proteins: NALCN, and TPM2, ANKK1. Our study provides valuable insights into the molecular mechanisms underlying CRC development.

## Graphical abstract

Endomucin (EMCN) was overexpressed in tumor tissues compared to normal para‐carcinoma tissues. overexpression of EMCN induced colorectal cancer proliferation and metastasis both in vitro and in vivo experiments. identified EMCN‐binding proteins were significantly enriched in carcinogenesis related functions and pathways.

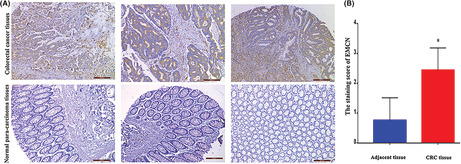

## 1 INTRODUCTION

Human colorectal cancer (CRC) is a common malignant tumor that causes a substantial burden worldwide.^1^, ^2^ In 2018, there were an estimated 1.8 million new cases of CRC, accounting for 10.2% of new cancer cases worldwide, and nearly 0.8 million deaths, accounting for 9.2% of global deaths.^3^, ^4^ Many lifestyle factors, such as smoking, alcohol consumption, being overweight or obese, and physical inactivity, contribute to an increased risk of CRC.^5^ Although there have been important advances in CRC treatment, such as surgery, radiotherapy, chemotherapy, targeted therapy, and immunotherapy,^6^, ^7^ the prognosis remains poor for patients with advanced CRC.^8^, ^9^ So a comprehensive understanding of the underlying molecular mechanisms as well as novel therapeutic strategies for the treatment of CRC are urgently needed.

Endomucin (EMCN), a type I integral membrane O‐sialoglycoprotein rich in serine and threonine residues, is an 80–120 kDa transmembrane sialoprotein that contains a long extracellular (aa 1–190), transmembrane (aa 191–214), and cytoplasmic (aa 215–261) domain.^10^, ^11^ EMCN is related to the cellular adhesion between vascular endothelial cells and neutrophils, where interference with its expression on quiescent endothelial cells promotes the adhesion of neutrophils to vascular endothelial cells; its expression during an inflammatory event prevents neutrophil adhesion and inhibits the infiltration of CD45+ and NIMP‐R14+ cells.^12^ Previous studies have demonstrated the importance of regulating cell–cell and cell–matrix interactions.^12^, ^13^ Furthermore, EMCN plays a key role in the proliferation and migration of vascular endothelial cells.^14^ Based on the importance of EMCN in vascular endothelial cells, it speculates that it may also play an important role in tumors. However, the role and mechanism of EMCN in tumors remain unclear.

Identifying the key factors and signal regulatory networks affecting the invasion and metastasis of CRC and screening treatment targets is important in improving the survival rate of patients with CRC. However, the current mechanism analysis of CRC is not comprehensive, and further exploration into its potential metastatic mechanism is necessary to lay a foundation for exploring new treatment options and drugs for CRC. Therefore, our study explored the role of EMCN in CRC and discussed its possible mechanism. Our results help to clarify the complex roles of EMCN in CRC and suggest that it may be a novel regulator of CRC development and that EMCN may be a potential therapeutic target for the treatment of CRC.

## 2 MATERIALS AND METHODS

### 2.1 Cell culture and transfection

The human CRC cell lines SW480, SW620, HCT116, HT29, and LoVo used in this study were cultured in RPMI 1640 medium (RPMI 1640; HyClone) supplemented with 10% fetal bovine serum (FBS, Gibco). All cell lines were incubated at 37°C in a humidified atmosphere containing 5% CO_2_. SW480 cells were infected with a lentivirus expressing EMCN or a corresponding control lentivirus (Shanghai Genechem Co.) at a multiplicity of infection (MOI) of 10. LoVo cells were infected with an EMCN‐knockdown lentivirus or a corresponding control lentivirus (Shanghai Genechem Co.) at an MOI of 50.

### 2.2 Mice

All SCID mice were housed and maintained under specific pathogen‐free conditions. All experiments were performed in accordance with the accepted national standards and guidelines. Six‐week‐old male Nu/Nu mice were used in all the experiments.

### 2.3 Tissue specimens

CRC and normal para‐carcinoma tissues were randomly collected, with prior approval, from patients who underwent surgical treatment at the First Affiliated Hospital of Chengdu Medical College. All procedures were approved by the Institutional Ethics Committee of Chengdu Medical College before commencing the study and all study participants provided informed consent. The tissue samples were immediately frozen in liquid nitrogen until further use.

### 2.4 Western blot analysis

Total protein from the CRC cell lines SW480, SW620, HCT116, HT29, and LoVo cells were separated by 10% SDS‐PAGE and transferred onto PVDF membranes. The following polyclonal primary antibodies were used: anti‐E‐cadherin (1:1000 dilution, AF6759, Beyotime), anti‐fibronectin (1:1000 dilution, 26836S, Cell Signaling Technology), anti‐β‐Catenin (1:1000 dilution, AF0066, Beyotime), anti‐EMCN (1:1000 dilution, PA5‐21395, Thermo Fisher Scientific), anti‐FLAG (1:1000 dilution, AF519, Beyotime), anti‐IgG (1:1000 dilution, A7016, Beyotime), anti‐β‐actin (1:4000 dilution, 20536‐1‐AP, Proteintech), and anti‐GAPDH (1:5000 dilution, 10494‐1‐AP, Proteintech). HRP‐conjugated anti‐rabbit IgG (1:3000, SA00001‐2, Proteintech) and HRP‐conjugated anti‐mouse IgG (1:2500 dilution, 7076S, Cell Signaling Technology) were used as secondary antibodies. The bands were visualized using an enhanced chemiluminescence system (Amersham Pharmacia Biotech).

### 2.5 Quantitative reverse transcription PCR

Total RNA was extracted from cultured cells (SW480, SW620, HCT116, HT29, and LoVo cells) using the TRIzol reagent (3101‐100, Shanghai Pufei Biotechnology). We then synthesized cDNA from the total RNA samples using an M‐MLV reverse transcription kit (M1705, Promega). Quantitative PCR was performed on the resulting cDNA samples using the SYBR Master Mix (DRR041B, TAKARA).

### 2.6 H&E staining

The histopathological characteristics of lung metastases in mice were evaluated using hematoxylin and eosin (H&E) staining (Beyotime Institute of Biotechnology, Inc.). The tissue specimens were fixed in 10% formalin and embedded in paraffin. Sections were prepared at a thickness of 4 μm and stained with H&E after dewaxing and hydration. After dehydration, transparency, and sealing, the sections were observed under an optical microscope.

### 2.7 Immunohistochemistry

Before immunostaining, the tumor tissue slides were deparaffinized, rehydrated, and steeped in 3% H_2_O_2_/PBS for 15 min to quench endogenous peroxidase. The tissue sections were then heated at 121°C in an autoclave for 10 min in 0.1 M citrate buffer (pH 6.0) to retrieve the antigens. After blocking the serum for 1 h, the sections were incubated with an anti‐EMCN antibody (1:1000 dilution, PA5‐21395, Thermo Fisher Scientific) and then with a secondary antibody, according to the manufacturer's instructions. Fresh DAB solution was added to each slice. Finally, the slides were counterstained with hematoxylin and the cover slipped.

Stained tissue sections were evaluated randomly by three independent blind observers according to immunoreactivity score (IRS). IRS was based on the percentage of positive cells (4, >80%; 3, 51%–80%; 2, 10%–50%; 1, <10%; 0, 0%) and staining intensity (3, strong; 2, moderate; 1, mild; and 0, no staining). IRS scores ranged from 0 to 12. EMCN expression levels were grouped into low (IRS <4) and high (IRS ≥4) expression levels.

### 2.8 CCK‐8 assay

Cell proliferation was evaluated using a CCK‐8 kit (MA0218, Dalian Meilun Biotechnology Co.). SW480 cells overexpressing transfected EMCN and LoVo cells with EMCN knocked down and their corresponding control cells were seeded in 96‐well plates at 2 × 10^3^ cells/well, with three replicate wells, and allowed to incubate for 48 h. The CCK‐8 reagent was added to each well and the cells were incubated for 2 h. The absorbance at 450 nm was measured using a microplate reader (Power WaveXS2; Biotek). All experiments were performed in triplicate, at least on three separate occasions.

### 2.9 Oris™ cell migration assay

The Oris™ cell migration assay (Platypus Technologies) was used to monitor cell migration in real time. LoVo cells with EMCN knockdown and corresponding control cells were grown to 90%–99% confluence were removed and resuspended to a final concentration of 3–5 × 10^6^/ml in FBS‐free media. Cells (100 μl) were plated in each well via the side port of the Oris™ Cell Seeding Stopper and allowed to adhere overnight in a humidified chamber (37°C, 5% CO_2_). The stoppers were then removed and a low‐serum medium (0.5% FBS) was added. The cells were incubated at 37°C for 8 or 48 h and images were captured using a fluorescence microscope (IX71, Olympus).

### 2.10 Transwell migration assays

Cell migration assays were performed using transwell plates. SW480 cells overexpressing transfected EMCN and LoVo cells with knocked down EMCN and their corresponding control cells were cultured in a fresh serum‐free medium in the upper chamber. Cells attached to the lower side were fixed in 4% paraformaldehyde and stained with 0.5% crystal violet. The migrated cells were counted in five microscopic fields.

### 2.11 Subcutaneous xenograft models

Xenograft tumors were established by the subcutaneous injection of CRC cells (5 × 10^6^) expressing high or low levels of EMCN. Tumor volume was measured with calipers and calculated according to the following formula: 0.5 × length × width^2^. After 6 weeks, all mice were anesthetized with isoflurane, and the tumors were removed, weighed, fixed, and embedded. The mice were euthanized at the end of the experiment. Changes in tumor volume and survival time were used as measures of tumorigenesis.

### 2.12 In vivo model of lung metastasis

To investigate lung metastasis, 3 × 10^6^ cells were injected into the tail vein of 6‐week‐old male Nu/Nu mice. Six weeks after tumor cell injection, the mice were anesthetized with isoflurane, and samples were collected. The mice were euthanized at the end of the experiment. Whole‐mount images of metastatic nodules in lung tissue were captured. Tissue samples were sectioned and subjected to H&E staining.

### 2.13 Co‐immunoprecipitation (Co‐IP)

IP lysate buffer (Cat. no. ab206996, Abcam) was used to obtain cell lysates (SW480 cells overexpressing transfected EMCN and corresponding control cells), and a BCA protein assay kit (Beyotime) was used to quantify protein concentration. Cell lysates of the same protein quality were cultured at 4°C overnight with either control IgG (Cat. no. A7016, Beyotime) or anti‐FLAG (Cat. no. AF519, Beyotime). The samples were then incubated with Protein A/G Sepharose Beads (Cat. no. ab206996; Abcam) at 4°C and mixed for 5 h. The mixture was centrifuged at 2000*g* for 2 min at 4°C. The beads were washed and resuspended in wash buffer. The proteins were reduced with 100 mM DTT for 5 min at 100°C following centrifugation at 2000*g* for 15 min, and the supernatant was stored at −80°C for mass spectrometric (MS) analysis.

### 2.14 MS analysis of EMCN complexes

The digested peptide mixtures were subjected to FASP enzymatic digestion. Following desalting, liquid chromatography–tandem mass spectrometry (LC–MS/MS) was performed using a Q‐Exactive mass spectrometer coupled with an Easy nLC (Thermo Fisher Scientific). The peptide sample was first loaded onto a C18‐reversed‐phase analytical column (Thermo Fisher Scientific, Acclaim PepMap RSLC 50 μm × 15 cm, nano viper, P/N164943) in buffer A (0.1% formic acid in high‐performance liquid chromatography grade water) and separated with a linear gradient of buffer B (80% acetonitrile and 0.1% formic acid) at a flow rate of 300 nL/min. A linear chromatographic gradient was achieved with a linear increase in buffer B percentage, which was set up as follows: 6% buffer B for 5 min, 6%–28% buffer B for 40 min, 28%–38% buffer B for 5 min, 38%–100% buffer B for 5 min, and hold in 100% buffer B for 5 min. The peptide was then added to a Q Exactive mass spectrometer (Thermo Fisher Scientific). MS analysis was performed for 60 min in positive ion mode.

### 2.15 PPI network analysis

The STRING database (https://string‐db.org)^15^ was used to search for interacting genes/proteins to analyze and visualize the protein–protein interaction (PPI) in the EMCN‐binding protein, utilizing the default confidence score.

### 2.16 Functional and pathway enrichment analyses

The DAVID database was used to perform gene ontology (GO) and Kyoto Encyclopedia of Genes and Genomes (KEGG) pathway enrichment analysis.^16^ The adjusted *p* < 0.05 was set as the selection criterion for significant GO and KEGG terms.

### 2.17 Statistical analyses

We used SPSS Statistics 21.0 to analyze the data. The chi‐square test and Fisher's exact probability method were used to analyze the relationship between EMCN and clinicopathological characteristics. A two‐tailed paired Student's *t*‐test was used to compare two groups. *p* values of <0.05 were considered a significant difference between the two groups. The results are expressed as the mean ± *SD*.

## 3 RESULTS

### 3.1 EMCN was upregulated in CRC tissues

**FIGURE 1:**
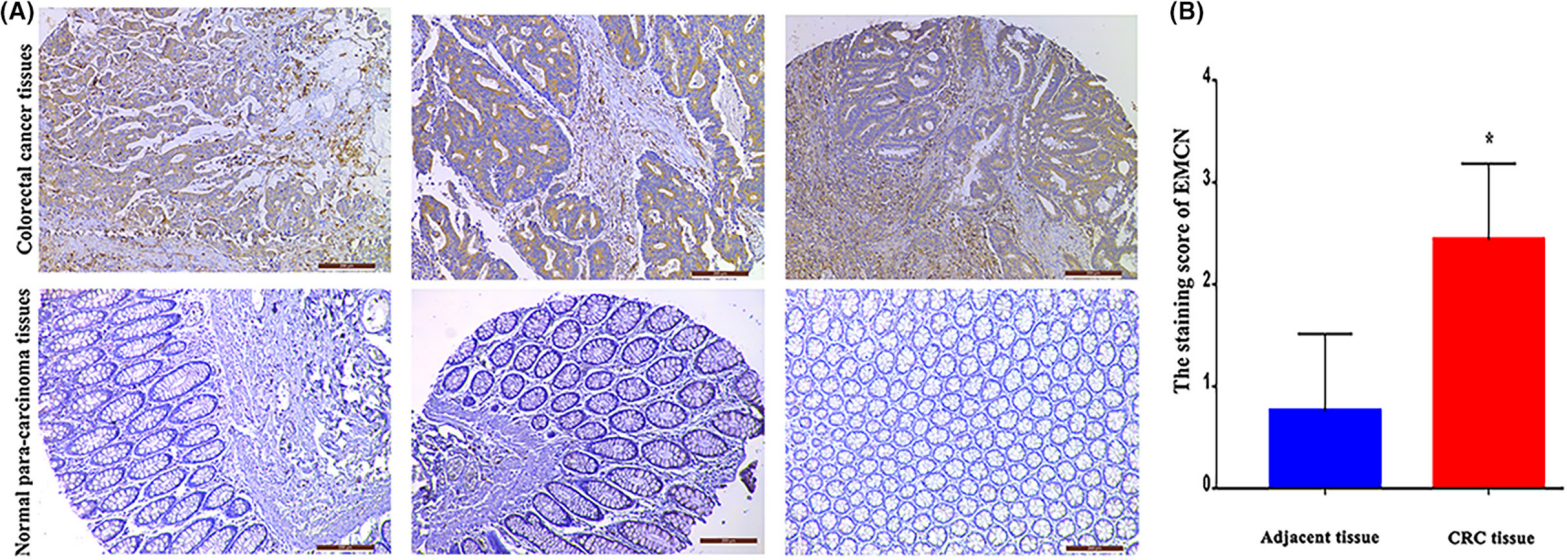
EMCN expression in CRC tissues and normal para‐carcinoma tissues. (A) Representative immunohistochemistry images of CRC as well as normal para‐carcinoma tissues are shown. The expression level of EMCN was scored by IRS and grouped into low (IRS <4) and high (IRS ≥4) expression levels. (B) The analysis of the expression of EMCN in CRC, including matched normal para‐carcinoma tissues. *p < 0.05 versus normal para‐carcinoma tissues. CRC, colorectal cancer; EMCN, endomucin; IRS, immunoreactivity score

EMCN expression was examined in CRC and normal para‐carcinoma tissues using immunohistochemistry. The analysis showed that EMCN expression levels were higher in CRC tissues than those in matched normal para‐carcinoma tissues from the same patient (Figure 1). The EMCN staining score was significantly lower in adjacent normal tissues than in CRC tissue samples (*p* < 0.05, Figure 1). Next, we analyzed the association between EMCN expression levels and the clinical features of patients with CRC. The levels of EMCN expression were significantly correlated with tumor differentiation (*p* = 0.007), N classification (*p* = 0.001), clinical stage (*p* = 0.006), and distant metastasis (*p* < 0.001) (Tables 1 and 2). Together, these findings provided strong evidence that EMCN was upregulated in CRC tissues and that the level of EMCN expression was related to the clinical characteristics of CRC.

**TABLE 1:** Statistical analysis of the relationship of EMCN expression with clinicopathologic characteristics in CRC patients

Clinicopathologic parameters	EMCN expression		*p* value
	Low	High	
Gender			
Male	33	44	0.617
Female	20	22	
Age			
<65	38	48	0.901
≥65	15	18	
Differentiation			
Well	11	9	**0.007**
Moderate	19	43	
Poor	21	13	
T classification			
T2	4	9	0.569
T3	34	40	
T4	15	17	
N classification			
N0	19	43	**0.001**
N1–N2	34	23	
Clinical stage			
I	3	7	**0.006**
II	16	36	
III–IV	34	23	

*P* values were calculated using chi‐square tests. Bold values *p* < 0.05 indicates a significant relationship among the variables.

Abbreviations: CRC, colorectal cancer; EMCN, endomucin.

**TABLE 2:** Statistical analysis of the relationship of EMCN expression with tumor size and distant metastasis in CRC patients

Clinicopathologic parameters	EMCN expression		*p* value
	Low	High	
Gender			
Male	15	51	0.252
Female	9	52	
Age			
<65	13	44	0.310
≥65	11	59	
Tumor size			
≤5 cm	7	59	0.267
>5 cm	1	31	
Distant metastasis			
No	8	91	**<0.001**
Yes	16	12	

*P* values were calculated using chi‐square tests. Bold values *p* < 0.05 indicates a significant relationship among the variables.

Abbreviations: CRC, colorectal cancer; EMCN, endomucin.

### 3.2 Construction of EMCN overexpression and interference cell lines

**FIGURE 2:**
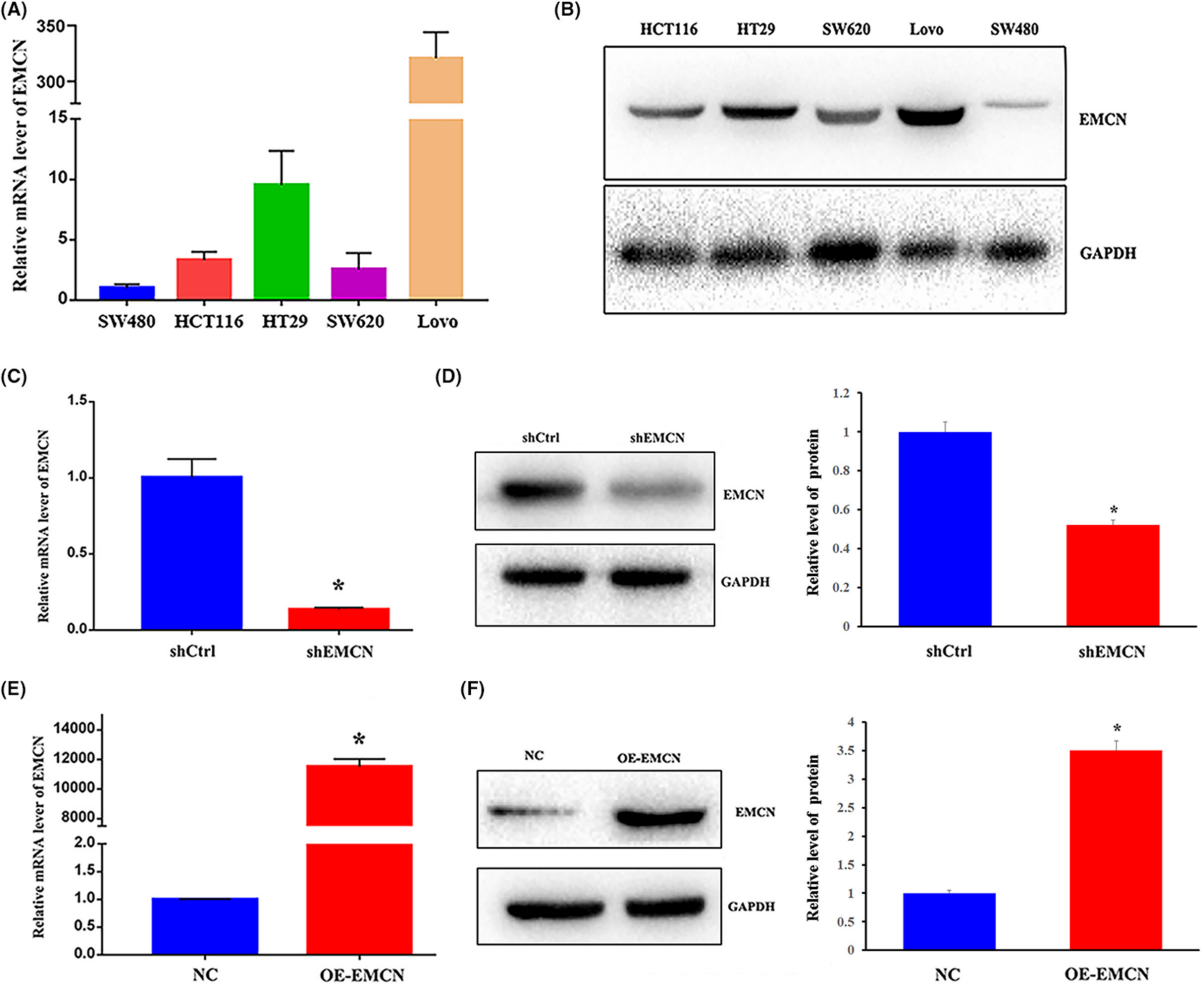
Construction of EMCN overexpression and interference cell lines. (A) EMCN mRNA expression was analyzed by RT‐PCR in CRC cell lines. (B) EMCN protein expression was analyzed via immunoblot in CRC cell lines. (C, E) RT‐PCR was utilized to test EMCN mRNA levels in EMCN‐knockdown (C), EMCN‐overexpressing (E) group. (D, F) Immunoblot was utilized to test EMCN protein in EMCN‐knockdown (D), EMCN‐overexpressing (F) group, and the histogram of the gray values of the relative EMCN protein expression levels were shown. Data represent the mean ± *SD*. *p < 0.05 versus control. CRC, colorectal cancer; EMCN, endomucin

To construct EMCN expression‐regulatory cells, EMCN expression was detected in CRC cell lines. SW480, SW620, HCT116, HT29, and LoVo cells were used to detect EMCN expression by RT‐PCR and western blotting. This indicated that EMCN was expressed in all CRC cell lines, with the highest expression levels in LoVo cells and the lowest in SW480 cells (Figure 2A,B). To examine the role of EMCN in the proliferation of CRC cells, we established a LoVo cell line with a stable knockdown of EMCN and an SW480 cell line overexpressing EMCN with a FLAG tag. RT‐PCR and western blotting were performed to determine whether the model cells were successfully constructed. The results showed that themRNA level of EMCN in interfering cells was significantly reduced, which was <20% of that noted in the control group (Figure 2C). And the protein expression level of EMCN in interfering cells was 52.2% of that in control cells (Figure 2D). The mRNA level of EMCN was more than 10,000 times higher than that in the control group (Figure 2E), and the protein expression level increased more than twice (Figure 2F). This indicated that EMCN knockdown and overexpression cell lines were successfully constructed.

### 3.3 EMCN promoted proliferation in vitro and in vivo

**FIGURE 3:**
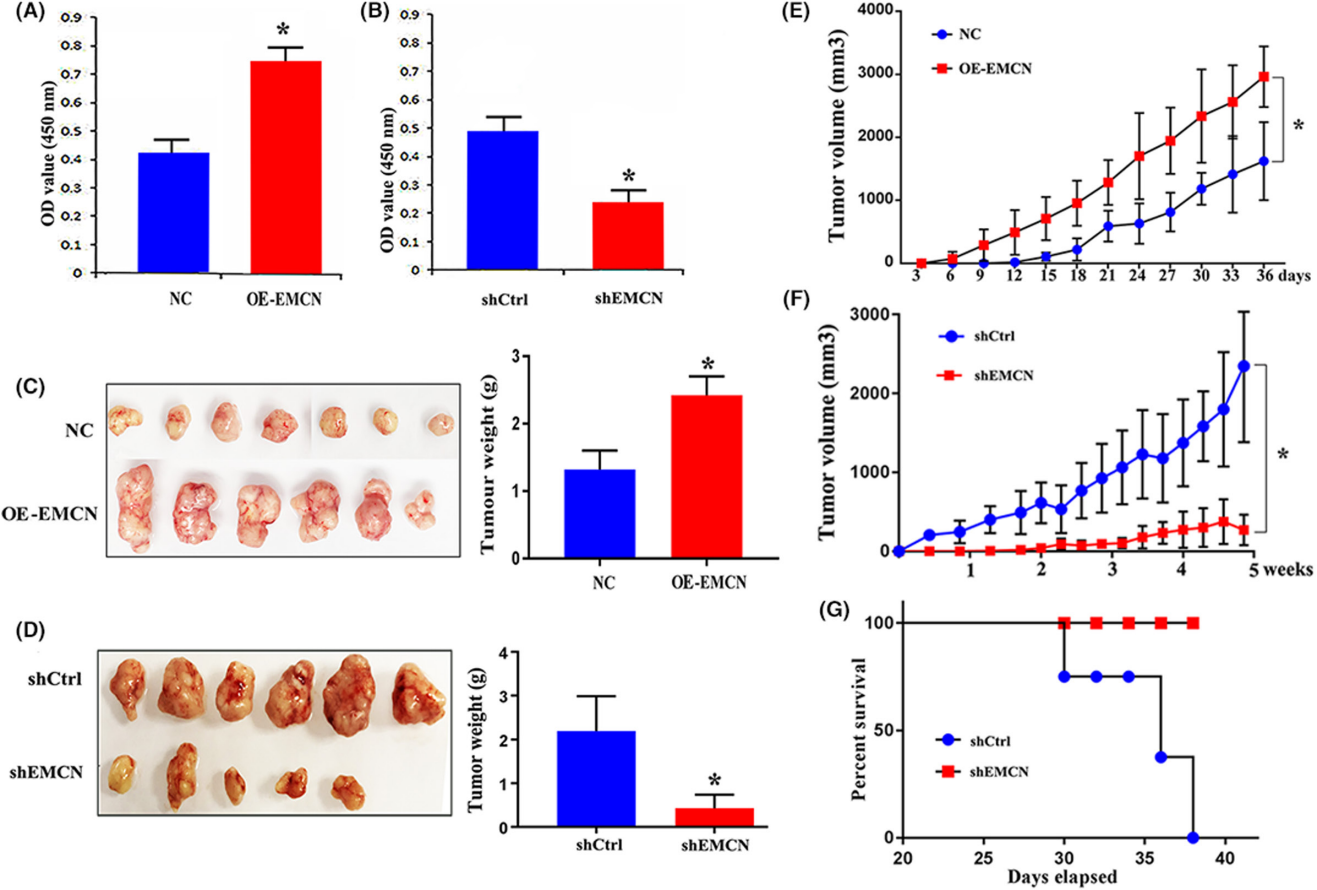
EMCN promoted CRC proliferation both in vitro and in vivo. (A, B) Cell proliferation was evaluated with CCK‐8 at 48 h. Data represent the mean ± *SD*. *p < 0.05 versus control. (C, D) Left: gross tumors in nude mice and right: tumor weight in nude mice. Data represent the mean ± *SD*. *p < 0.05 versus control. (E, F) Tumor growth curves. Data represent the mean ± *SD*. *p < 0.05 versus control. (G) The percent survival of the two groups of nude mice injected with EMCN‐knockdown or control cells. CRC, colorectal cancer; EMCN, endomucin

CCK‐8 assays were used to measure the proliferation of EMCN knockdown and overexpression cell lines. The proliferation of SW480 cells overexpressing EMCN was considerably higher than that of control cells (Figure 3A). However, the proliferation of LoVo cells with EMCN knockdown was significantly suppressed compared with that of control cells (Figure 3B).

To further validate the oncogenic effect of EMCN on the proliferation of CRC cells in vivo, we performed tumorigenesis experiments in nude mice. The results of the subcutaneous tumor model experiment showed that the tumor volume growth rate in the EMCN overexpression group was considerably higher than that in the control group. At the experimental cutoff point, the tumor volume and weight of the overexpression group were markedly higher than those of the control group. During the experiment, one mouse in the overexpression group died because of excessive tumor load (Figure 3C,E); however, only five mice in the EMCN‐knockdown group grew slow‐growing tumors. At the experimental cutoff point, the average tumor volume in the EMCN‐knockdown group was very small, approximately 0.2 cm^3^, while all six mice in the control group grew tumors that grew rapidly (Figure 3D,F). Thirty‐eight days after cell inoculation, all tumors in the control group exceeded 1.5 cm^3^ and were determined dead. Whereas the tumor volumes in the EMCN‐knockdown group were still small (did not exceed 1.5 cm^3^) 42 days postinoculation and were considered alive (Figure 3G). In conclusion, these data indicated that EMCN is associated with CRC tumorigenesis, both in vitro and in vivo.

### 3.4 EMCN promoted CRC metastasis

**FIGURE 4:**
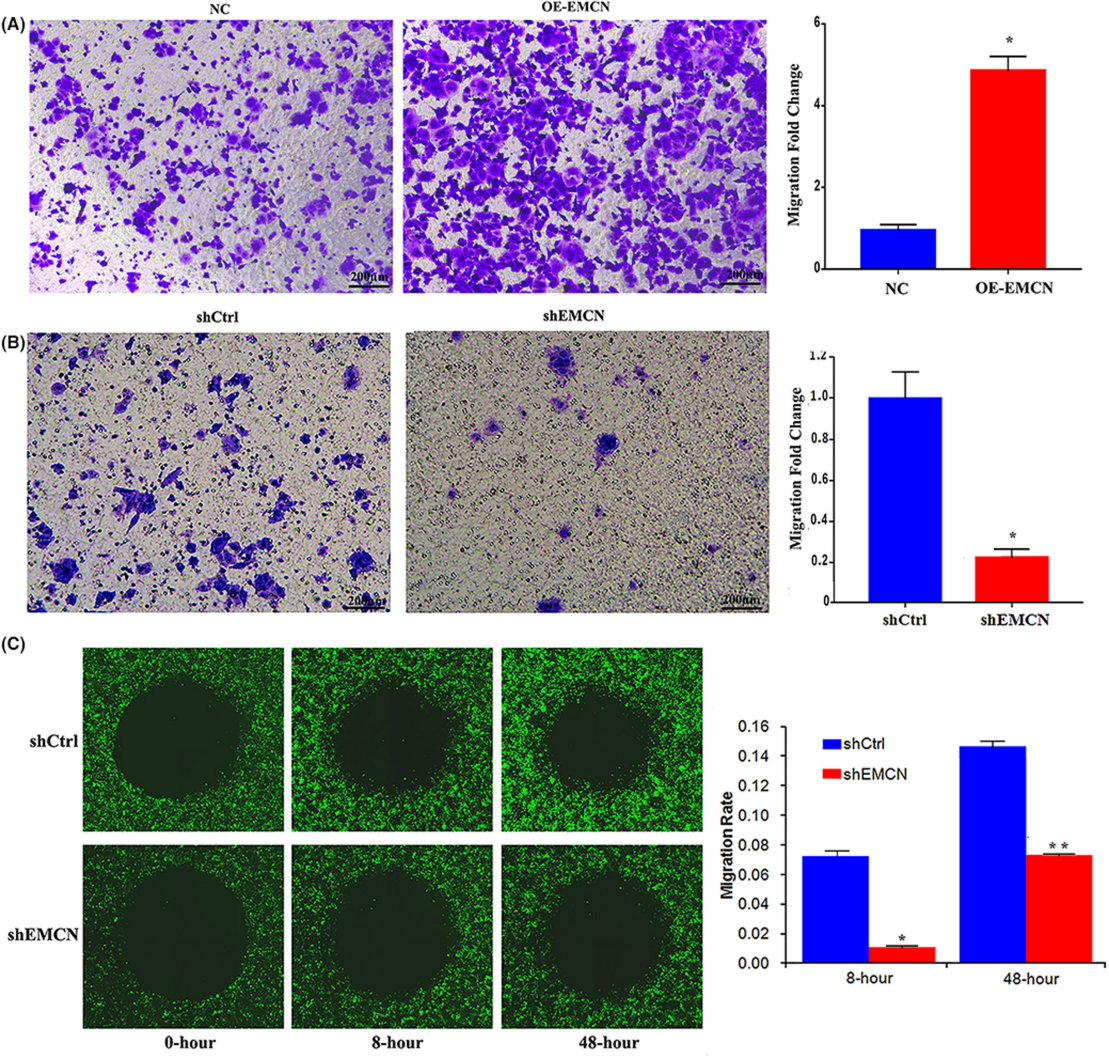
Endomucin (EMCN) promoted colorectal cancer metastasis in vitro. (A, B) Representative images from the Transwell migration assay in EMCN‐knockdown, EMCN‐overexpressing cells, and histogram showing the migration fold change in each group. Scale: 200 μm. Data represent the mean ± *SD*. *p < 0.05 versus control. (C) ORIS™ cell migration assay in EMCN knockdown, and histogram showing the migration rate in each group. Data represent the mean ± *SD*. *p < 0.05 versus control. **p < 0.01 versus control

Transwell and ORIS™ cell migration assays were used to determine the effect of EMCN on CRC cell migration in vitro. Figure 4A shows that EMCN overexpression significantly promoted the migration of CRC cell lines compared with the control cell lines. However, EMCN knockdown had the opposite effect (Figure 4B). Consistent with these findings, data from the ORIS™ cell migration assay system indicated that cell migration was impaired when EMCN was knocked down (Figure 4C). Taken together, these data suggested that EMCN was important for modulating CRC cell migration in vitro.

**FIGURE 5:**
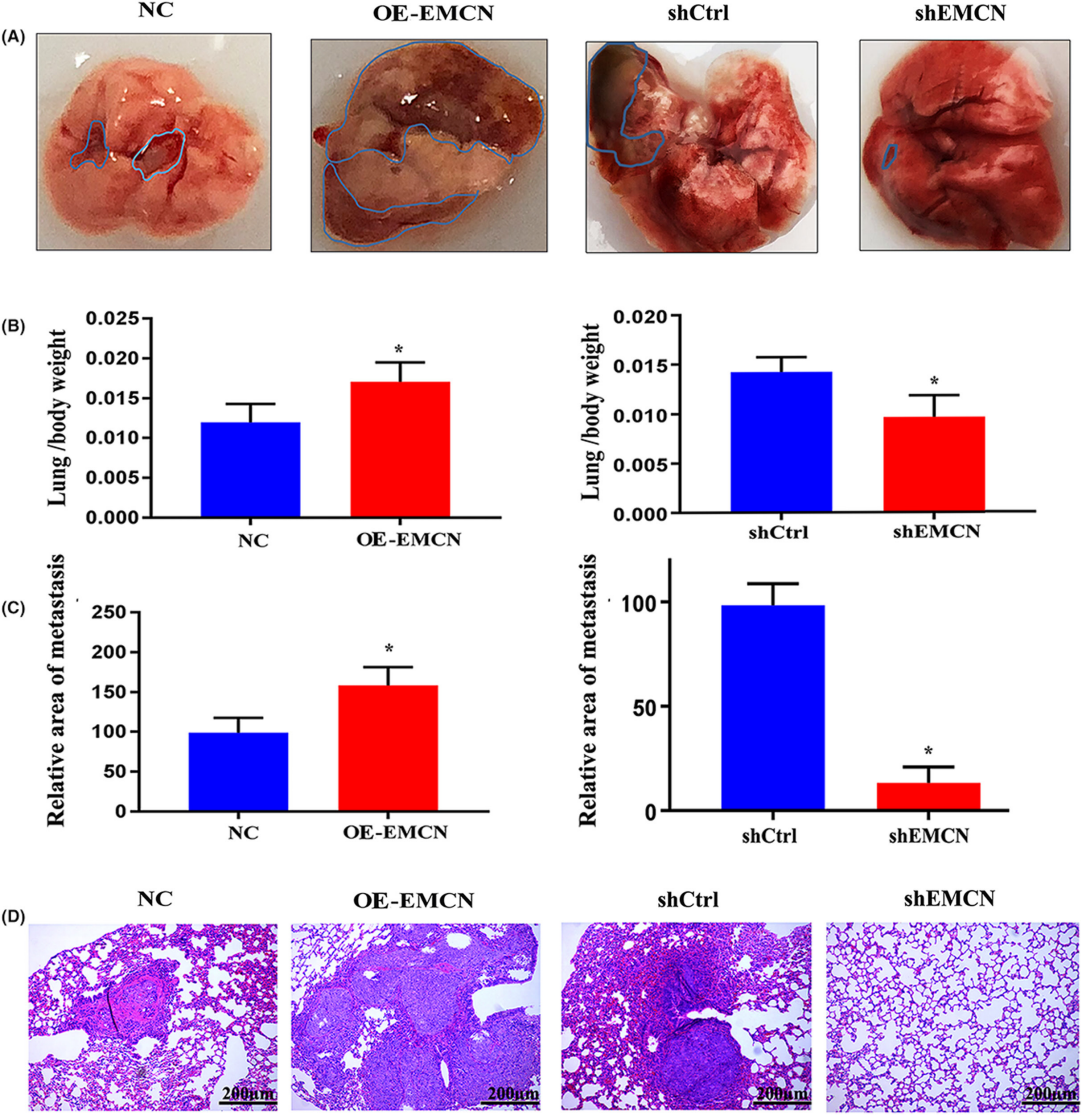
EMCN promotes CRC lung metastasis in vivo. (A) Representative images of lung metastasis of EMCN‐overexpressing cells and EMCN knockdown and their respective control cells in vivo. (B) The ratio of the lung to body weight. Data represent the mean ± *SD*. *p < 0.05 versus control. (C) The relative area of lung metastasis. Data represent the mean ± *SD*. *p < 0.05 versus control. (D) Paraffin‐embedded tumor tissues were generated in the lung metastasis model subjected to H&E staining. CRC, colorectal cancer; EMCN, endomucin; H&E, hematoxylin and eosin

To determine whether EMCN is involved in CRC metastasis in vivo, we generated a lung metastasis model using nude mice. As shown in Figure 5A, larger metastatic nodules were observed in the EMCN overexpression group, but smaller metastatic nodules were observed in the EMCN‐knockdown group compared with the control group. Furthermore, the ratio of lung weight to body weight was considerably higher, and the relative area of metastasis was markedly greater in the EMCN overexpression group than in the control group, whereas the opposite effects were observed in the EMCN‐knockdown group (Figure 5B,C). H&E staining of mice lungs showed that only one mouse in the EMCN interference group developed lung metastasis, while all six mice in the control group developed tumor lung metastasis. H&E staining revealed irregular lung cell arrangement in mice injected with EMCN‐overexpressed cells. Moreover, no contours were detected, and there was no clear distinction between the nucleus and cytoplasm of the cells. However, the opposite outcome was observed with EMCN knockdown (Figure 5D). These results indicated that EMCN promoted CRC metastasis in vivo.

### 3.5 EMCN played a key role in EMT

**FIGURE 6:**
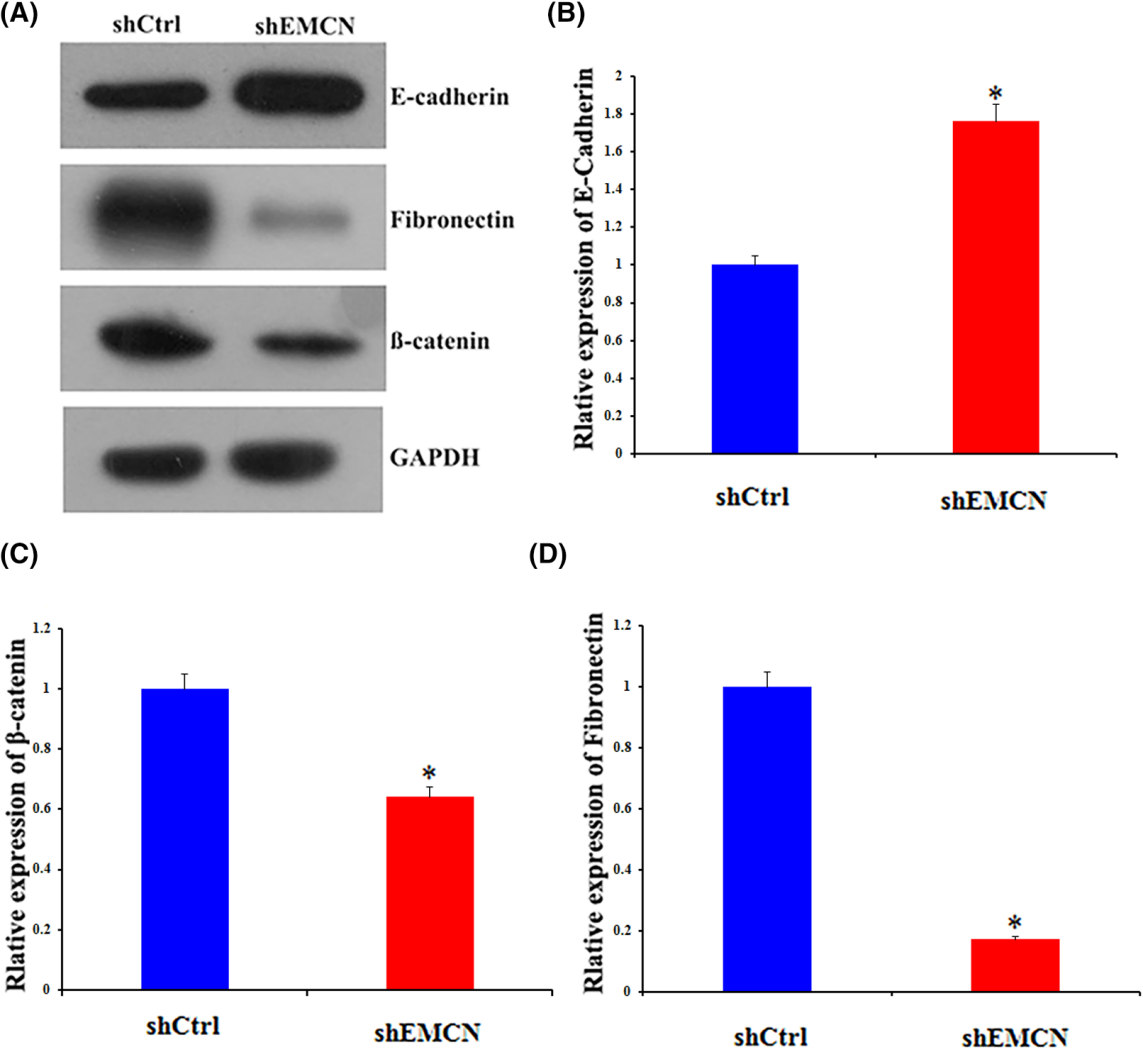
EMCN regulated EMT in CRC cell lines. (A) E‐cadherin, β‐catenin, and fibronectin levels in EMCN‐knockdown cells and control cells were determined by western blotting. (B–D) The histogram of the gray values shows the relative protein expression levels of E‐cadherin (B), β‐catenin (C), fibronectin (D) in EMCN‐knockdown cells. Data represent the mean ± *SD*. *p < 0.05 versus control. CRC, colorectal cancer; EMCN, endomucin; EMT, epithelial–mesenchymal transition

Epithelial–mesenchymal transition (EMT) is a key characteristic of tumor infiltration and metastasis. It is characterized at the molecular level by the upregulation of mesenchymal markers, such as fibronectin and β‐catenin, and the downregulation of epithelial differentiation markers, such as E‐cadherin. The expression of E‐cadherin in EMCN‐knockdown cells is 1.76‐fold that of the control cells (Figure 6A,B). However, the protein expression level of β‐catenin in EMCN‐knockdown cells was 64% of that noted in control cells (Figure 6A,C), and the protein expression level of fibronectin in EMCN‐knockdown cells was 17.3% of that noted in control cells (Figure 6A,D). Together, these data provided strong evidence that EMCN played a key role in the EMT in CRC.

### 3.6 Identification and functional annotation of potential EMCN‐binding proteins

**FIGURE 7:**
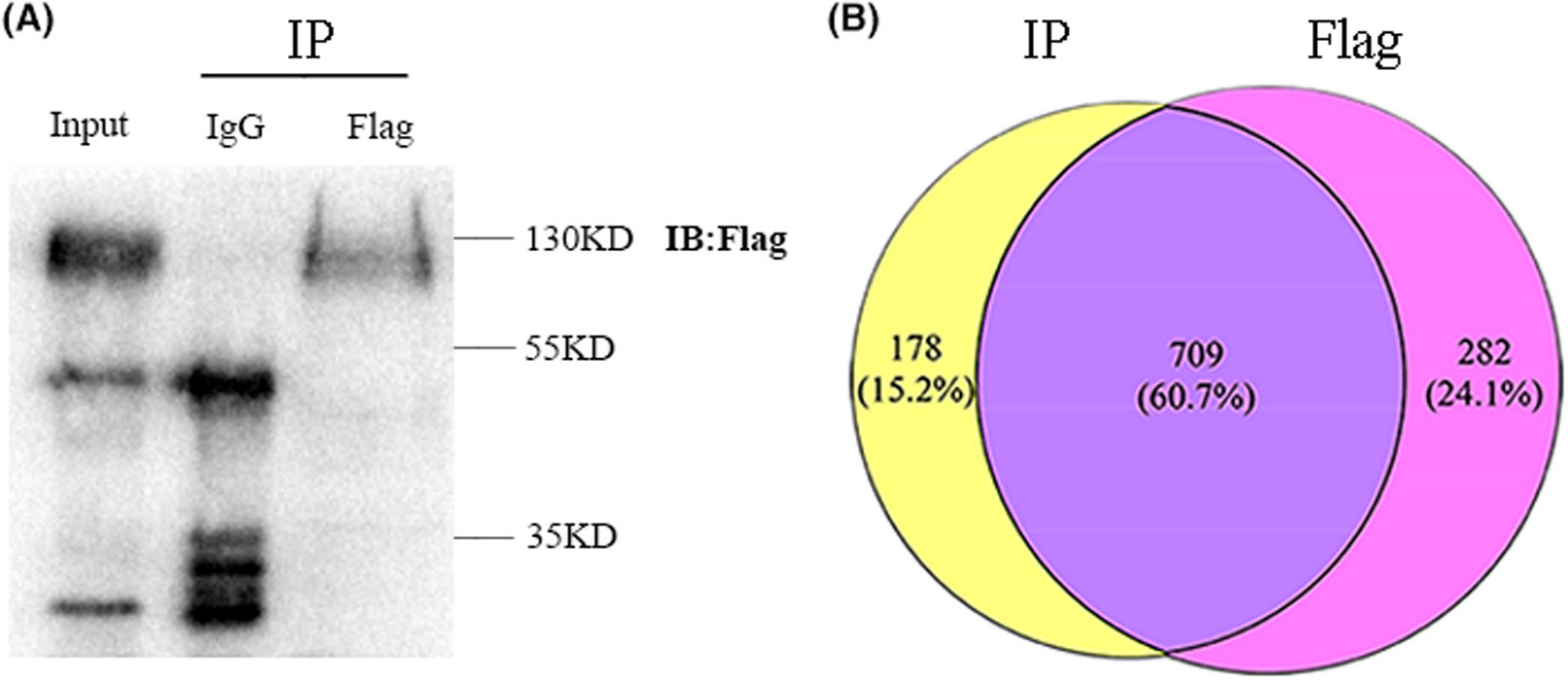
The results of potential EMCN‐interacting proteins by Co‐IP and LC–MS/MS. (A) Potential EMCN‐interacting proteins were immunoprecipitated under EMCN‐overexpressing SW480 cells lysate using anti‐FLAG antibodies and then blotted with an anti‐FLAG antibody. The input is shown in lane 1; the EMCN‐overexpressing SW480 cells lysate that has been immunoprecipitated with normal IgG is shown as a negative control in lane 2; and the immunoprecipitation with the specified antibody in lane 3. (B) Venn diagram showing the potential EMCN‐interacting proteins identified by LC–MS/MS after CO‐IP of EMCN‐overexpressing cells with a flag tag. Co‐IP, coimmunoprecipitation; EMCN, endomucin; IgG, immunoglobulin; LC–MS/MS, liquid chromatography–tandem mass spectrometry

The results showed that EMCN was critical for the proliferation and metastasis of CRC, but the mechanism remains unclear. We want to study the interacting proteins of EMCN in CRC, so as to analyze the mechanism of EMCN affecting the progression of CRC. To determine EMCN protein partners, we performed a proteomic study using Co‐IP of EMCN‐overexpressing cells with a FLAG tag (Figure 7A). Co‐IP experiments were conducted in triplicate, followed by LC–MS/MS to identify potential EMCN interactors. Venn analysis revealed 178 potential EMCN‐binding proteins (Figure 7B), and the merged list of identified potential EMCN‐interacting proteins from Co‐IP and MS is shown in Table S1.

**FIGURE 8:**
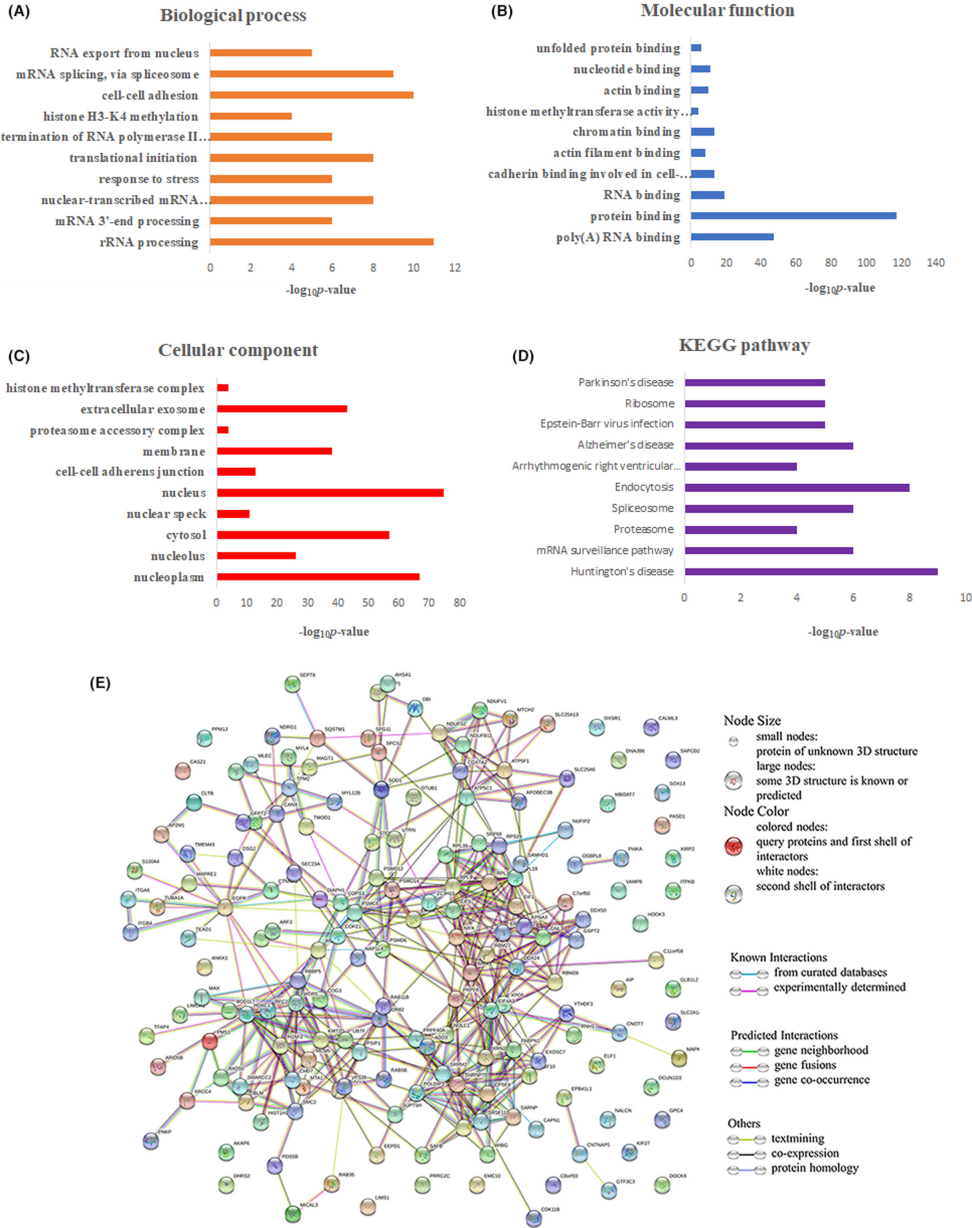
Functional and pathway terms rich in potential EMCN‐binding partners. (A–C) GO term enrichment of the potential EMCN‐binding partners according to the biological process (A), molecular function (B), and cellular component (C). (D) KEGG pathway analysis of the potential EMCN‐binding proteins. (E) PPI network of potential EMCN‐binding partners. PPI visualization networks by String 11.5 using 168 potential EMCN‐binding proteins. EMCN, endomucin; GO, gene ontology; KEGG, Kyoto Encyclopedia of Genes and Genomes; PPI, protein–protein interaction

A functional enrichment analysis was performed to determine the functions and pathways of potential EMCN‐binding proteins. The results indicated potential EMCN‐binding proteins mainly enriched in GO biological processes included “cell–cell adhesion,” “rRNA processing,” “mRNA splicing, via spliceosome,” “nuclear‐transcribed mRNA catabolic process, nonsense‐mediated decay,” and “translational initiation” (Figure 8A). The main enriched GO molecular functions included “protein binding,” “poly(A) RNA binding,” “RNA binding,” “cadherin binding involved in cell–cell adhesion,” and “chromatin binding” (Figure 8B). The main enriched GO cellular components included the “nucleolus,” “nucleoplasm,” “cytosol,” “extracellular exosome,” and “membrane” (Figure 8C). The KEGG pathway analysis showed EMCN‐binding proteins may be related to “Huntington's disease,” “Endocytosis,” “mRNA surveillance pathway,” “Spliceosome,” and “Alzheimer's disease” (Figure 8D). Moreover, PPI network analysis showed that EMCN partners were highly interconnected (Figure 8E).

### 3.7 Screening for potential EMCN‐binding proteins related to the prognosis of CRC

**FIGURE 9:**
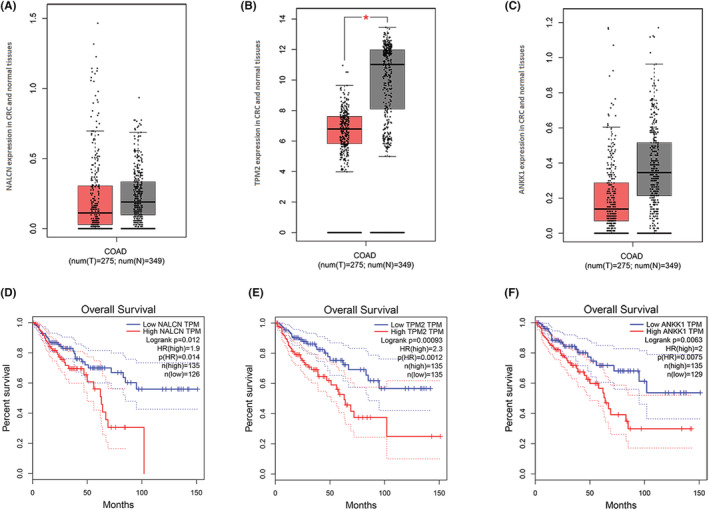
Expression levels of NALCN (A), TPM2 (B) and ANKK1 (C) in CRC and normal samples from the GEPIA database. Expression levels are shown as box plots and scatter plots. Survival analysis of NALCN (D), TPM2 (E) and ANKK1 (F) in CRC from the GEPIA database. The high and low expression groups are indicated as red and blue curves, respectively. ANKK1, ankyrin repeat and kinase domain containing 1; NALCN, sodium leak channel; TPM2, tropomyosin‐2

To identify the binding proteins related to the carcinogenic function of EMCN, we performed expression and Kaplan–Meier analysis using the GEPIA database.^17^ We screened out proteins that were differentially expressed in cancer and normal para‐carcinoma tissues, and these proteins were related to tumor prognosis. We then used Hum‐mPLoc 3.0^18^ to query the subcellular location of the candidate protein in the cytoplasm or cell membrane. Subcellular localization showed that the sodium leak channel (NALCN) was located in the plasma membrane, Tropomyosin‐2 (TPM2), and ankyrin repeat and kinase domain containing 1 (ANKK1) were located in Cytoplasm. The results showed that NALCN, TPM2, and ANKK1 were differentially expressed in CRC (Figure 9A–C), and their expression was related to the poor prognosis of CRC (Figure 9D–F). Therefore, EMCN may bind to ANKK1, NALCN, and TPM2 to promote CRC development.

## 4 DISCUSSION

In this study, we found that EMCN was expressed in CRC and promoted proliferation and metastasis. As metastasis is the primary cause of death in cancer patients, up to one‐fourth of CRC patients have metastatic disease at the time of diagnosis, with the most commonly affected sites being the liver, lungs, and peritoneum.^19^, ^20^ EMCN, which is expressed on the surfaces of venules, capillaries, and lymphatic vessels, is an integral membrane glycocalyx glycoprotein.^21^, ^22^ Its abundant O‐glycans attach to serine and threonine residues and maintain a highly rigid and extended structure that regulates cell–cell and cell–matrix interactions.^13^ A previous study indicated that EMCN overexpression leads to decreased leukocyte adhesion to the retinal vessel wall in streptozotocin‐induced diabetic rats.^23^ However, the mechanism of action of EMCN in tumors is unclear. Our study showed that EMCN can promote the progression of CRC, providing a theoretical basis for improving the regulatory mechanism of CRC progression and providing experimental data for screening CRC metastasis markers and therapeutic targets.

We found that EMCN knockdown upregulated E‐cadherin expression and downregulated β‐catenin and fibronectin expression, indicating that the EMT process was inhibited. The mechanisms underlying metastasis are complicated and involve a multistep process from the site of origin to distant organs.^19^ It has been reported that EMT, a process by which cells gradually lose their epithelial phenotype and obtain a mesenchymal phenotype, is a key developmental program that is often activated during cancer invasion and metastasis.^24^, ^25^, ^26^ After activating EMT, cancer cells exhibit a molecular shift, characterized by decreased expression levels of epithelial markers, such as E‐cadherin, and increased expression levels of mesenchymal markers, such as β‐catenin, N‐cadherin, and fibronectin.^24^, ^26^, ^27^ Our research showed that EMCN played a key role in EMT in CRC.

These results showed that EMCN was critical for the proliferation and metastasis of CRC, but the mechanism remains unclear. We believe that EMCN‐binding proteins may be important for EMCN function and play an important role in CRC development and progression. Using Co‐IP and LC–MS/MS, 178 EMCN‐binding proteins were identified. After an in‐depth analysis of these EMCN‐binding proteins, it was observed that NALCN, TPM2, and ANKK1 were differentially expressed and correlated with poor CRC prognosis. TPM2 is a member of the tropomyosin gene family and is closely related to cytoskeletal functions, such as cytokinesis, vesicle transport, cell proliferation, migration, and apoptosis, and is related to the occurrence of cancer. TPM2 is closely related to paclitaxel resistance in breast cancer patients, with poor survival rates.^28^ Studies have shown that high‐grade, recurrent, and metastatic prostate tumors express lower TPM2 than intermediate, nonrecurrent, and primary prostate tumors.^29^ The Hum‐mPLoc 3.0 database predicts that TPM2 is in the cytoplasm. NALCN is a voltage‐independent Na + channel that is expressed in many cancers,^30^ such as pancreatic cancer, nonsmall‐cell lung cancer, tumor‐derived endothelial cells, and glioblastoma. NALCN is involved in many processes, such as locomotive behavior and sensitivity to volatile anesthetics.^31^ The Hum‐mPLoc 3.0 database predicts that NALCN is in the plasma membrane, and the GEPIA database indicates that NALCN is differentially expressed in CRC tissues and related to tumor prognosis. ANKK1 is a member of the receptor‐interacting protein kinase family and plays a key role in cell survival and death, cell proliferation, differentiation, and gene transcription.^32^, ^33^, ^34^ Therefore, we speculate that TPM2, NALCN, and ANKK1 may interact with EMCN and play a carcinogenic role. But their comprehensive function of them needs to be considered in the complex context of CRC. We have only preliminarily screened EMCN‐interacting proteins without experimental verification. Future studies will validate EMCN‐interacting proteins and explore downstream regulatory mechanisms.

In this study, we found that EMCN overexpression promoted CRC proliferation and metastasis, whereas EMCN knockdown inhibited CRC proliferation, metastasis, and the EMT process. Furthermore, we performed a comprehensive proteomics analysis of Co‐IP and LC–MS/MS, which resulted in the identification of 178 EMCN‐binding proteins. Functional annotation analysis indicated that EMCN‐binding proteins were rich in oncogenic functions and pathways. We initially screened for three EMCN‐binding proteins, NALCN, TPM2, and ANKK1, but further research is necessary to validate and extend our findings.

## 5 CONCLUSIONS

Our results showed that EMCN was overexpressed in CRC tissues. Moreover, our data also demonstrated that overexpression of EMCN induced proliferation and promoted CRC metastasis both in vivo and in vitro. Furthermore, EMCN silencing inhibited EMT. We identified 178 EMCN‐binding proteins and initially screened three potential EMCN‐interacting proteins: NALCN, TPM2, and ANKK1. Our study provides valuable insights into the molecular mechanisms underlying CRC development.

## AUTHOR CONTRIBUTIONS

Yan Zhou conceived and designed the study. Qi Huang performed the experiments and collected the data. Xue‐mei Li and Jing‐ping Sun analyzed and interpreted the data. Yan Zhou, Xue‐mei Li, and Qi Huang prepared the manuscript. All the authors read and approve the final manuscript.

## FUNDING INFORMATION

The present study was supported by the Scientific Research Foundation of Mianyang Central Hospital (grant no. 2021YJRC‐002).

## CONFLICT OF INTEREST

The authors declare that they have no conflict of interest.

## ETHICS STATEMENT

All animal experiments were performed in accordance with relevant guidelines and regulations and approved by the Institutional Animal Care and Use Committee of Chengdu Medical College. For the use of these clinical materials for research purposes, prior patient consent and approval from the Institutional Ethics Committee of Chengdu Medical College were obtained.

## CONSENT FOR PUBLICATION

Not applicable.

## Supporting information


Table S1

## Data Availability

The data sets used and/or analyzed during the current study are available from the corresponding author upon reasonable request.

## References

[1] Sharma R . An examination of colorectal cancer burden by socioeconomic status: evidence from GLOBOCAN 2018. EPMA J. 2020;11(1):95‐117. 32140188 10.1007/s13167-019-00185-y PMC7028897

[2] Pilleron S , Sarfati D , Janssen‐Heijnen M , et al. Global cancer incidence in older adults, 2012 and 2035: a population‐based study. Int J Cancer. 2019;144(1):49‐58. 29978474 10.1002/ijc.31664

[3] Bray F , Ferlay J , Soerjomataram I , Siegel RL , Torre LA , Jemal A . Global cancer statistics 2018: GLOBOCAN estimates of incidence and mortality worldwide for 36 cancers in 185 countries. CA Cancer J Clin. 2018;68(6):394‐424. 30207593 10.3322/caac.21492

[4] Ferlay J , Colombet M , Soerjomataram I , et al. Estimating the global cancer incidence and mortality in 2018: GLOBOCAN sources and methods. Int J Cancer. 2019;144(8):1941‐1953. 30350310 10.1002/ijc.31937

[5] Gu M‐J , Huang Q‐C , Bao C‐Z , et al. Attributable causes of colorectal cancer in China. BMC Cancer. 2018;18(1):1‐9. 29304763 10.1186/s12885-017-3968-z PMC5756355

[6] Kavousipour S , Khademi F , Zamani M , Vakili B , Mokarram P . Novel biotechnology approaches in colorectal cancer diagnosis and therapy. Biotechnol Lett. 2017;39(6):785‐803. 28238060 10.1007/s10529-017-2303-8

[7] Ganesh K , Stadler ZK , Cercek A , et al. Immunotherapy in colorectal cancer: rationale, challenges and potential. Nat Rev Gastroenterol Hepatol. 2019;16(6):361‐375. 30886395 10.1038/s41575-019-0126-x PMC7295073

[8] Tauriello DV , Calon A , Lonardo E , Batlle E . Determinants of metastatic competency in colorectal cancer. Mol Oncol. 2017;11(1):97‐119. 28085225 10.1002/1878-0261.12018 PMC5423222

[9] Siegel RL , Miller KD , Fedewa SA , et al. Colorectal cancer statistics, 2017. CA Cancer J Clin. 2017;67(3):177‐193. 28248415 10.3322/caac.21395

[10] Liu C , Shao Z‐M , Zhang L , et al. Human endomucin is an endothelial marker. Biochem Biophys Res Commun. 2001;288(1):129‐136. 11594763 10.1006/bbrc.2001.5737

[11] Kinoshita M , Nakamura T , Ihara M , et al. Identification of human endomucin‐1 and‐2 as membrane‐bound O‐sialoglycoproteins with anti‐adhesive activity. FEBS Lett. 2001;499(1–2):121‐126. 11418125 10.1016/s0014-5793(01)02520-0

[12] Zahr A , Alcaide P , Yang J , et al. Endomucin prevents leukocyte–endothelial cell adhesion and has a critical role under resting and inflammatory conditions. Nat Commun. 2016;7(1):1‐10. 10.1038/ncomms10363 PMC4740757 26831939

[13] Hanisch F‐G . O‐glycosylation of the mucin type. 2001. 10.1515/BC.2001.022 11308013

[14] Park‐Windhol C , Ng YS , Yang J , Primo V , Saint‐Geniez M , D'Amore PA . Endomucin inhibits VEGF‐induced endothelial cell migration, growth, and morphogenesis by modulating VEGFR2 signaling. Sci Rep. 2017;7(1):1‐13. 29215001 10.1038/s41598-017-16852-x PMC5719432

[15] Szklarczyk D , Franceschini A , Wyder S , et al. STRING v10: protein–protein interaction networks, integrated over the tree of life. Nucleic Acids Res. 2015;43(D1):D447‐D452. 25352553 10.1093/nar/gku1003 PMC4383874

[16] Huang DW , Sherman BT , Tan Q , et al. DAVID bioinformatics resources: expanded annotation database and novel algorithms to better extract biology from large gene lists. Nucleic Acids Res. 2007;35(suppl_2):W169‐W175. 17576678 10.1093/nar/gkm415 PMC1933169

[17] Tang Z , Li C , Kang B , Gao G , Li C , Zhang Z . GEPIA: a web server for cancer and normal gene expression profiling and interactive analyses. Nucleic Acids Res. 2017;45(W1):W98‐W102. 28407145 10.1093/nar/gkx247 PMC5570223

[18] Zhou H , Yang Y , Shen H‐B . Hum‐mPLoc 3.0: prediction enhancement of human protein subcellular localization through modeling the hidden correlations of gene ontology and functional domain features. Bioinformatics. 2017;33(6):843‐853. 27993784 10.1093/bioinformatics/btw723

[19] Lambert AW , Pattabiraman DR , Weinberg RA . Emerging biological principles of metastasis. Cell. 2017;168(4):670‐691. 28187288 10.1016/j.cell.2016.11.037 PMC5308465

[20] Lee RM , Cardona K , Russell MC . Historical perspective: two decades of progress in treating metastatic colorectal cancer. J Surg Oncol. 2019;119(5):549‐563. 30806493 10.1002/jso.25431

[21] LeBlanc ME , Saez‐Torres KL , Cano I , et al. Glycocalyx regulation of vascular endothelial growth factor receptor 2 activity. FASEB J. 2019;33(8):9362‐9373. 31141406 10.1096/fj.201900011R PMC6662976

[22] Kuhn A , Brachtendorf G , Kurth F , et al. Expression of endomucin, a novel endothelial sialomucin, in normal and diseased human skin. J Invest Dermatol. 2002;119(6):1388‐1393. 12485444 10.1046/j.1523-1747.2002.19647.x

[23] Niu T , Zhao M , Jiang Y , et al. Endomucin restores depleted endothelial glycocalyx in the retinas of streptozotocin‐induced diabetic rats. FASEB J. 2019;33(12):13346‐13357. 31545913 10.1096/fj.201901161R

[24] Dongre A , Weinberg RA . New insights into the mechanisms of epithelial–mesenchymal transition and implications for cancer. Nat Rev Mol Cell Biol. 2019;20(2):69‐84. 30459476 10.1038/s41580-018-0080-4

[25] Mittal V . Epithelial mesenchymal transition in tumor metastasis. Annu Rev Pathol. 2018;13:395‐412. 29414248 10.1146/annurev-pathol-020117-043854

[26] Prieto‐Garcia E , Díaz‐García CV , García‐Ruiz I , Agulló‐Ortuño MT . Epithelial‐to‐mesenchymal transition in tumor progression. Med Oncol. 2017;34(7):1‐10. 28560682 10.1007/s12032-017-0980-8

[27] Buczek M , Miles A , Green W , et al. Cytoplasmic PML promotes TGF‐β‐associated epithelial–mesenchymal transition and invasion in prostate cancer. Oncogene. 2016;35(26):3465‐3475. 26549027 10.1038/onc.2015.409 PMC4932557

[28] Zhang J , Zhang J , Xu S , et al. Hypoxia‐induced TPM2 methylation is associated with chemoresistance and poor prognosis in breast cancer. Cell Physiol Biochem. 2018;45(2):692‐705. 29414807 10.1159/000487162

[29] Varisli L . Identification of new genes downregulated in prostate cancer and investigation of their effects on prognosis. Genet Test Mol Biomarkers. 2013;17(7):562‐566. 23621580 10.1089/gtmb.2012.0524

[30] Djamgoz MB . Hyponatremia and cancer progression: possible association with sodium‐transporting proteins. Bioelectricity. 2020;2(1):14‐20. 34471833 10.1089/bioe.2019.0035 PMC8370306

[31] Cochet‐Bissuel M , Lory P , Monteil A . The sodium leak channel, NALCN, in health and disease. Front Cell Neurosci. 2014;8:132. 24904279 10.3389/fncel.2014.00132 PMC4033012

[32] Meylan E , Tschopp J . The RIP kinases: crucial integrators of cellular stress. Trends Biochem Sci. 2005;30(3):151‐159. 15752987 10.1016/j.tibs.2005.01.003

[33] Rubio‐Solsona E , Martí S , Vílchez JJ , Palau F , Hoenicka J . ANKK1 is found in myogenic precursors and muscle fibers subtypes with glycolytic metabolism. Plos One. 2018;13(5):e0197254. 29758057 10.1371/journal.pone.0197254 PMC5951577

[34] Fedorenko OY , Paderina DZ , Loonen AJ , et al. Association of ANKK1 polymorphism with antipsychotic‐induced hyperprolactinemia. Hum Psychopharmacol. 2020;35(4):e2737. 32383805 10.1002/hup.2737 PMC7507142

